# Changes at the nuclear lamina alter binding of pioneer factor Foxa2 in aged liver

**DOI:** 10.1111/acel.12742

**Published:** 2018-02-27

**Authors:** Holly Whitton, Larry N. Singh, Marissa A. Patrick, Andrew J. Price, Fernando G. Osorio, Carlos López‐Otín, Irina M. Bochkis

**Affiliations:** ^1^ Broad Institute of MIT and Harvard Cambridge MA USA; ^2^ Center for Mitochondrial and Epigenomic Medicine Children's Hospital of Philadelphia Philadelphia PA USA; ^3^ Department of Pharmacology University of Virginia Charlottesville VA USA; ^4^ Departamento de Bioquímica y Biología Molecular Facultad de Medicina Instituto Universitario de Oncología (IUOPA) Universidad de Oviedo Oviedo Spain; ^5^ Centro de Investigación Biomédica en Red de Cáncer Madrid Spain

**Keywords:** forkhead factors, Foxa2, heterochromatin, lipid metabolism, liver, nuclear lamina

## Abstract

Increasing evidence suggests that regulation of heterochromatin at the nuclear envelope underlies metabolic disease susceptibility and age‐dependent metabolic changes, but the mechanism is unknown. Here, we profile lamina‐associated domains (LADs) using lamin B1 ChIP‐Seq in young and old hepatocytes and find that, although lamin B1 resides at a large fraction of domains at both ages, a third of lamin B1‐associated regions are bound exclusively at each age in vivo. Regions occupied by lamin B1 solely in young livers are enriched for the *forkhead* motif, bound by Foxa pioneer factors. We also show that Foxa2 binds more sites in *Zmpste24* mutant mice, a progeroid laminopathy model, similar to increased Foxa2 occupancy in old livers. Aged and *Zmpste24*‐deficient livers share several features, including nuclear lamina abnormalities, increased Foxa2 binding, de‐repression of PPAR‐ and LXR‐dependent gene expression, and fatty liver. In old livers, additional Foxa2 binding is correlated to loss of lamin B1 and heterochromatin (H3K9me3 occupancy) at these loci. Our observations suggest that changes at the nuclear lamina are linked to altered Foxa2 binding, enabling opening of chromatin and de‐repression of genes encoding lipid synthesis and storage targets that contribute to etiology of hepatic steatosis.

## 1 INTRODUCTION

Increasing evidence suggests that regulation of heterochromatin at the nuclear envelope is a common mechanism underlying metabolic disease susceptibility and age‐dependent metabolic changes (Lopez‐Otin, Galluzzi, Freije, Madeo & Kroemer, 2016). A recent report implicated disorganization of heterochromatin at the lamina as a driver of human aging (Zhang et al., 2015). Mutations in *LMNA*, encoding the nuclear structural protein lamin A/C, result in disturbed nuclear architecture and cause the premature aging syndrome Hutchinson‐Gilford progeria (HGPS). Additionally, *LMNA* mutations lead to partial lipodystrophy, a condition associated with insulin‐resistant diabetes, hypertriglyceridemia, and hepatic steatosis (Shackleton et al., 2000). Multiple enzymes modulating covalent modifications to lysine 9 of histone 3 (H3K9), the mark associated with heterochromatin in lamina‐associated domains (Guelen et al., 2008), have been linked to fatty liver, hyperlipidemia, diabetes, and obesity (Picard et al., 2002; Sun et al., 2012; Tateishi, Okada, Kallin & Zhang, 2009; Villeneuve et al., 2008; Wang et al., 2013). We have recently implicated lamina‐associated factors Hdac3 and Srf in age‐dependent dysregulation of lipid metabolism in the liver (Bochkis, Przybylski, Chen & Regev, 2014). However, the mechanism relating chromatin disorganization at the nuclear lamina to metabolic defects is unknown.

Foxa2 is a member of the Foxa subfamily of winged‐helix/*forkhead* box (Fox) transcription factors comprised of three unlinked genes (*Foxa1, Foxa2, and Foxa3*) that share a highly conserved DNA‐binding domain (Friedman & Kaestner, 2006). The structure of Foxa *forkhead* box has been solved and resembles that of H1 histone (Clark, Halay, Lai & Burley, 1993). Foxa1 can bind and open compacted chromatin in vitro (Cirillo et al., 2002), while Foxa2 binds nucleosomal DNA in vivo (Li, Schug, Tuteja, White & Kaestner, 2011) and mediates nucleosomal depletion during differentiation (Li et al., 2012). Hence, Foxa proteins have been labeled as “pioneer” factors for their ability to bind highly condensed chromatin first, displacing linker histones, and enable access for subsequent binding of additional transcription factors (Zaret & Carroll, 2011). We also found that Foxa2 occupies considerably more regions in aged fatty liver, binding regions of decreased nucleosome occupancy at PPAR targets, and cooperating with PPAR receptors in regulation of gene expression changes that contribute to steatosis (Bochkis et al., 2014).

As we have suggested a relationship between lamina‐associated factors and age‐dependent decline of hepatic lipid metabolism (Bochkis et al., 2014), we set out to profile LADs using lamin B1 ChIP‐Seq to discover different genomic regions located at the nuclear lamina in young and old hepatocytes in an unbiased manner. While nuclei in young mice have a round shape, those in old mice exhibit nuclear lamina abnormalities (Andrew, Brown & Johnson, 1943; Jin et al., 2010). We find that although a large fraction of regions is bound by lamin B1 at both ages, a third of the domains are occupied by lamin B1 exclusively at each age. Regions bound by lamin B1 only in the young are enriched for the *forkhead* motif, bound by Foxa pioneer factors. Moreover, we show that binding of Foxa2 is increased in a progeroid laminopathy model, similar to increased Foxa2 occupancy in old livers, contributing to etiology of fatty liver and age‐dependent metabolic dysfunction. In old livers, additional Foxa2 binding is correlated to loss of lamin B1 and heterochromatin (H3K9me3 occupancy) at these loci. Together, our observations suggest that changes at the nuclear lamina are linked to altered Foxa2 binding, enabling opening of chromatin and de‐repression of genes encoding lipid synthesis and storage targets that contribute to development of hepatic steatosis.

## 2 RESULTS

### 2.1 Lamin B1 binds distinct domains in young and old livers

**Figure 1:**
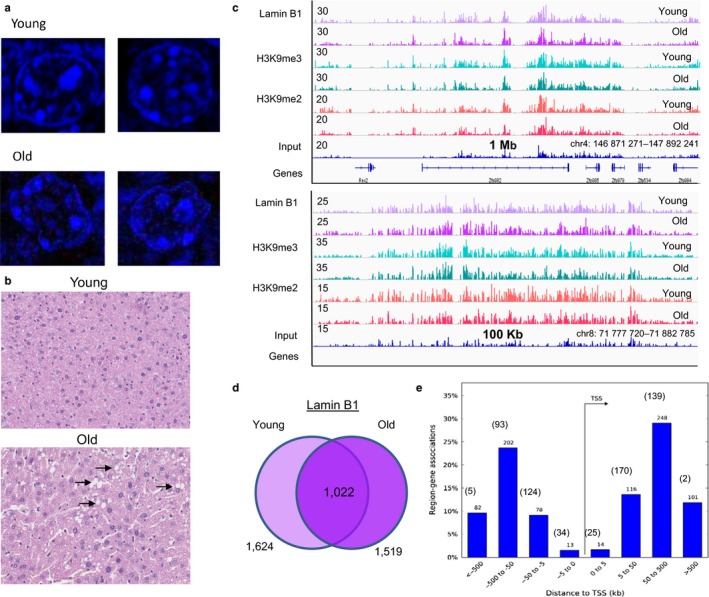
Lamin B1 binds distinct domains in young and old livers. (a) Nuclear immunofluorescence staining (Hoechst 33,258, 1:2,000) of liver sections from young and old mice. Nuclei in young livers have a round shape (top panel), while nuclei in old livers are irregular in shape and distorted (bottom panel). (b) Representative liver sections from young and old mice stained with hematoxylin and eosin (H&E). Lipid accumulation is apparent on histological sections by presence of lipid droplets in old livers (arrows, bottom panel). (c) ChIP‐Seq track view in Integrative Genome Viewer (IGV) of common lamina‐associated domains (LADs) found in both young and old wild‐type (WT) male livers, profiled by binding of lamin B1, and H3K9me2 and H3K9me3 chromatin marks (top panel: chr4:146,871,271‐147,892,241, bottom panel: chr8:71,777,720‐71,882,785). Reads are merged from two replicates in each condition. Track of input reads is provided for comparison. Size of the region shown on *x*‐axis and magnitude of ChIP‐Seq signal is shown on *y*‐axis. (d) Venn diagram showing the results of genomewide location analysis for lamin B1 in young and old liver, identifying 1,624 domains in young and 1,519 in old, of which 1,022 were called bound in both young and old livers by Epic. (e) The distribution of sites occupied by lamin B1 (in young and old livers) around transcription start site (TSS). Values for young and old (in parentheses) for each bin (0 to 5 kb, 5 to 50 kb, 50 to 500 kb, and >500 kb) from TSS are shown above each bar. The majority of regions are distal, 5 to 500 kb from the TSS

As we have previously connected two lamina‐associated factors Hdac3 and Srf to dysregulation of lipid metabolism in aged liver (Bochkis et al., 2014), we decided to continue our investigation into the role of the nuclear lamina in metabolic dysfunction by profiling LADs in young and old livers using lamin B1 ChIP‐Seq (chromatin immunoprecipitation followed by sequencing, data merged from two replicates in each condition). Consistent with previous reports, nuclei in old livers are distorted and of irregular shape (Figure 1a, bottom panel), and aged hepatocytes accumulate lipids (Figure 1b, bottom panel, lipid droplets indicated by arrows). Lamina‐associated domains lack active histone marks and contain repressive H3K9me2 and H3K9me3 modifications (Guelen et al., 2008; Sadaie et al., 2013). Lamina‐associated domains present in both young and old livers, enriched for chromatin marks H3K9me2 and H3K9me3, are shown in Figure 1c. We computed pairwise correlations for the replicates (Pearson R) that showed that replicates were similar (lamin B1 – 0.78 for young, 0.66 for old; H3K9me3 – 0.78 for young, for 0.79 old; H3K9me2 – 0.65 for young, for 0.64 old). Epic peak caller, a more efficient implementation of SICER domain‐calling algorithm (Xu, Grullon, Ge & Peng, 2014), was used to ascertain lamin B1 occupancy. We found a similar number of lamin B1 domains in young and old hepatocytes (regions called by Epic, Figure 1d, 1,624 in young and 1,519 in old) in vivo. Although a large fraction of regions is bound by lamin B1 at both ages, a third of lamin B1‐associated regions are bound exclusively at each age. Binding sites were mapped to nearby genes using GREAT (McLean et al., 2010). Majority of regions were located distally, 5 to 500 kb from the transcription start site (TSS) in both conditions (Figure 1e). A list of genes embedded within LADs at each age is provided in Table S1. We compared our results (LADs in young livers) with previously defined LADs in mice (Peric‐Hupkes et al., 2010). The overlap ranged from 42% with mESC data to 47% with MEF LADs. The overlap is reasonable considering reported genomewide correlation of 0.6 between mESC and other cell types (Peric‐Hupkes et al., 2010) and differences between DamID and ChIP‐Seq methods.

**Figure 2:**
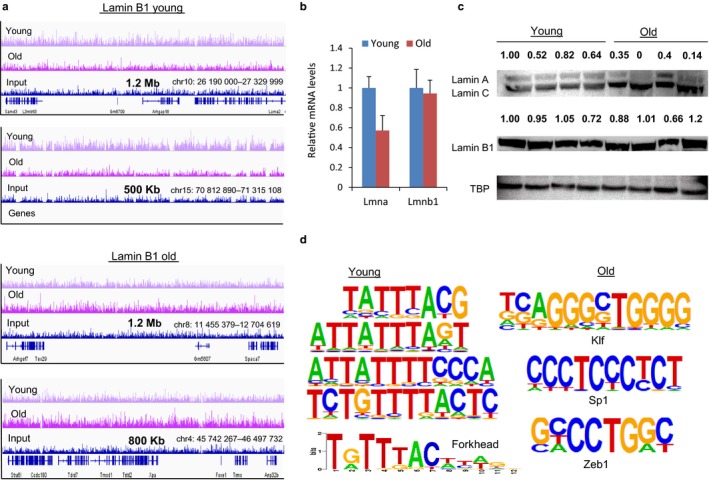
Changes in lamin B1 binding are independent of lamin B1 expression. (a) Examples of genomic regions bound by lamin B1 only in young (top panels: chr10:26,190,000‐27,329,999 and chr15:70,812,890‐71,315,108) and exclusively in old hepatocytes (bottom panels: chr8:11,455,379‐12,704,619 and chr4:45,742,267‐46,497,732) with input track as a comparison. Size of the region is shown on *x*‐axis and magnitude of ChIP‐Seq signal is shown on *y*‐axis. (b) mRNA levels of *Lmna*, gene that codes for lamin A and lamin C proteins, and Lmnb1, gene that codes for lamin B1 by quantitative RT–PCR. While mRNA levels of *Lmna* are downregulated about twofold (*t* test *p*‐value <.01), expression of *Lmnb1* is not changed (c) Western blot analysis of protein nuclear extracts from four young (3 months) and four old (21 months) mouse livers with antibodies to lamin A/C, lamin B1, TATA box‐binding protein (TBP, loading control). Although protein levels of lamin B1 also did not change, protein levels of lamin A declined in aged livers (about threefold, *t* test *p*‐value <.005). Protein expression is quantified by ImageJ and expression of both lamin A and lamin B1 is normalized to loading control TBP. (d) Multiple motifs resembling the *forkhead* (Fox) consensus (*p*‐values ranging from 1 × 10^−35^ to 1 × 10^−21^) were overrepresented in sites bound by lamin B1 in young livers, while Klf (*p*‐value 1 × 10^−43^), Sp1 (*p*‐value 1 × 10^−26^), and Zeb1 (*p*‐value 1 × 10^−35^) motifs were highly enriched in old livers

Next, we focused on genes in regions that were differentially bound by lamin B1 in either young or old livers (examples shown in Figure 2a.). We performed functional analysis of genes bound by lamin B1 only in young livers using Ingenuity Pathway Analysis (IPA). Pathways including “genes regulated by PPARα” (*p*‐value 1.1 × 10^−2^), PPARα/RXRα activation (*p*‐value 1.1 × 10^−2^), and TR/RXR activation (*p*‐value 2.3 × 10^−5^) were enriched among these targets. This observation is consistent with our previous report that Foxa2 binds regions of reduced nucleosome occupancy at PPARα targets in aged liver, leading to their activation (Bochkis et al., 2014). “liver proliferation” (*p*‐value 4.1 × 10^−3^), known to be impaired in aged liver (Timchenko, 2009), and “xenobiotic metabolism signaling” (*p*‐value 7.4 × 10^−3^) were pathways enriched in lamin B1‐associated regions in old livers. The full set of pathways for regions bound by lamin B1 is provided in Table S2.

To eliminate the possibility that changes in lamin B1 binding were due to differences in expression levels, we performed gene expression and protein analysis of lamina‐associated proteins. While mRNA levels of *Lmna*, gene that codes for lamin A and lamin C proteins, are downregulated about twofold (*t* test *p*‐value <.01), expression of *Lmnb1*, gene that codes for lamin B1 protein, is not changed in old livers (Figure 2b). Although protein levels of lamin B1 also did not change, confirming that changes in lamin B1 binding were independent of lamin B1 expression, protein levels of lamin A declined in aged livers (about threefold, *t* test *p*‐value <.005 expression of both lamin A and lamin B1 is normalized to loading control TBP, Figure 2c)

To identify potential regulators with differential occupancy in lamin B1 regions in young and old livers, we completed de novo motif analysis using HOMER (Heinz et al., 2010). LADs are A/T‐rich (Meuleman et al., 2013); hence, using a common genomic background (without a sequence bias) for both conditions to find motifs in lamin B1‐associated regions would not be appropriate. Thus, we compared sequences in lamin B1 domains against each other (young vs. old and old vs, young) to find motifs that were enriched in each condition. Multiple motifs resembling the *forkhead* (Fox) consensus (*p*‐values ranging from 1 × 10^−35^ to 1 × 10^−21^) were overrepresented in sites bound by lamin B1 in young livers (Figure 2d, left panel). In addition, motifs for Sox (*p*‐value 1 × 10^−27^) and GATA factors (*p*‐value 1 × 10^−25^) were enriched in these regions. Forkhead and GATA factors are established pioneer factors (Zaret & Carroll, 2011), which actively open chromatin by binding nucleosomal DNA first and enable subsequent binding of other factors, while Sox factors may also exhibit pioneering ability (Sarkar & Hochedlinger, 2013). The motifs for these regulators are found in regions bound by lamin B1 exclusively in young hepatocytes and possibly occupied by these pioneer factors in the old hepatocytes. In contrast, CG‐rich motifs for Klf factors (*p*‐value 1 × 10^−43^), Sp1 (*p*‐value 1 × 10^−26^), and Zeb1 (*p*‐value 1 × 10^−35^) motifs were highly enriched in lamin B1 regions bound in old livers (Figure 2d right panel). The motifs enriched in lamin B1 sites that are shared between young and old livers resemble young sites in their sequence profile. We have identified similar motifs by HOMER (forkhead motif *p*‐value 1 × 10^−6^, Sox *p*‐value 1 × 10^−7^, GATA *p*‐value 1 × 10^−3^), although enrichment is less significant than for regions bound by lamin B1 exclusively in young livers.

### 2.2 Foxa2 occupies more sites in Zmpste24 mutant livers

**Figure 3:**
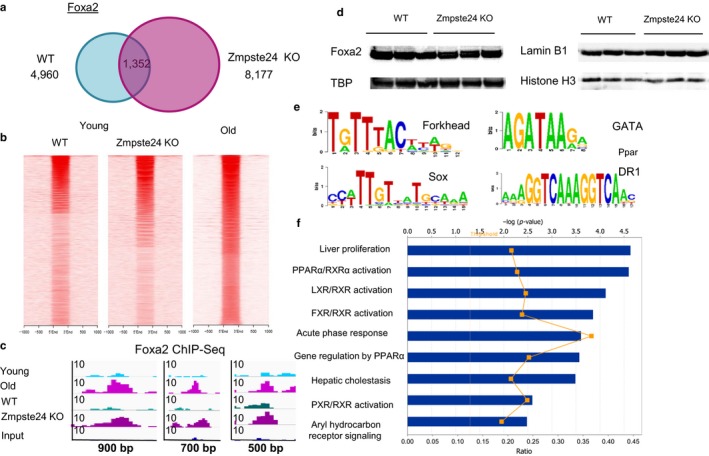
Foxa2 occupies more sites in *Zmpste24* mutant livers. (a) Venn diagram comparing Foxa2 binding in WT and *Zmpste24*‐deficient livers in male mice, 4,960 binding sites in wild‐type controls, and 8,177 in the mutants, of which 1,352 were called bound by both factors by PeakSeq. Data merged from three replicates in each condition. (b) Heatmaps comparing Foxa2 ChIP coverage in young wild‐type controls (left panel) and *Zmpste24* mutants (middle panel) of sites bound by Foxa2 in aged livers (right panel). (c) Examples of regions where Foxa2 binding in *Zmpste24*‐deficient livers (data merged from three replicates in each condition) resembles Foxa2 occupancy in old livers (left panel: chr11:120,206,031‐120,206,930, middle panel: chr18:54,980,906‐54,981,605, right panel: chr15:81,839,701‐81,840,205). Data for young and old livers (merged from two replicates in each condition) are from our previous study (Bochkis et al., 2014). Size of the region is shown on *x*‐axis and magnitude of ChIP‐Seq signal is shown on *y*‐axis. (d) Western blot analysis of protein nuclear extracts from three wild‐type controls and three *Zmpste24* mutant mouse livers (all 12 week old) with antibodies to FOXA2 and TATA box‐binding protein (TBP, loading control, left panel) and antibodies to LAMIN B1 and histone H3 (loading control, right panel). (e) Motifs for forkhead (*p*‐value 5.9 × 10^−110^), Sox (*p*‐value 7.6 × 10^−100^), GATA factors (*p*‐value 4.0 × 10^−67^), as well as DR‐1 element bound by PPAR nuclear receptors, were enriched in sequences associated with Foxa2 binding exclusively in *Zmpste24* male mutants. (f) Ingenuity Pathway Analysis (IPA) of genes associated with sites occupied by Foxa2 in *Zmpste24*‐deficient male livers shows that functional categories associated with activation of nuclear receptors FXR, LXR, PXR, and PPARα are enriched in these targets. –log (*p*‐value) is shown on *x*‐axis

Winged‐helix transcription factor Foxa2 plays an important role in lipid homeostasis in aged liver, binding regions of decreased nucleosome occupancy near PPAR‐dependent lipid synthesis and storage genes and contributing to gene expression changes that lead to steatosis (Bochkis et al., 2014). We have shown that Foxa2 occupies twice as many sites in older hepatocytes (6605 sites in young, 12,834 sites in old)(Bochkis et al., 2014), where lamin A expression is decreased (Figure 2b,c). In addition, forkhead motif is enriched in regions bound by lamin B1 only in young livers (Figure 2d). Hence, we hypothesized that increased Foxa2 binding in older livers is due to nuclear lamina defects accompanying aging and examined Foxa2 binding in a laminopathy mouse model. Zmpste24 is a metalloprotease that processes prelamin A to a mature form. *Zmpste24* mutants, similar to old mice, exhibit nuclear lamina abnormalities and develop fatty liver (Varela et al., 2005). We found that Foxa2 binding is increased in *Zmste24*‐deficient mice (8,177) compared to their wild‐type control littermates (4,960) (data merged from three replicates in each condition, peaks called by PeakSeq (Rozowsky et al., 2009), Figure 3a). We computed pairwise correlations for the replicates (Pearson R) that showed that replicates were similar (0.89, 0.85, 0.86 for WT, 0.74, 0.75, 0.81 for Zmpste24 KO). Comparing Foxa2 ChIP coverage in young wild‐type controls (Figure 3b, left panel) and *Zmpste24* mutants (Figure 3b, middle panel) of sites bound by Foxa2 in aged livers (Figure 3b, right panel), we found that more of these sites were bound in young *Zmpste24* mutants. Quantitative analysis (details in Experimental Procedures) of overlap between sites bound by Foxa2 in Zmpste24 mutants and old livers is shown in Figure S1. We find a large overlap as well as distinct regions bound by Foxa2 exclusively in Zmpste24 mutants or only in old livers. Some observed differences could be due to different circadian regulation in old mice and Zmpste24 mutants (Hood & Amir, 2017). Regions where Foxa2 binding in *Zmpste24*‐deficient livers resembles Foxa2 occupancy in old livers (Bochkis et al., 2014) are shown in Figure 3c. To eliminate the possibility that differences in Foxa2 binding were due to changes in expression levels, we measured Foxa2 protein levels in Zmpste24 mutants and their control littermates, and found that Foxa2 levels do not change (Figure 3d). Also, lamin B1 protein expression did not change in Zmpste24 mutant livers (Figure 3d).

Motif analysis in Foxa2 sites bound exclusively in *Zmpste24*‐deficient mice (positional weight matrix (PWM) scan using PscanChiP (Zambelli, Pesole & Pavesi, 2013)) identified consensus sequences for forkhead (*p*‐value 5.9 × 10^−110^), Sox (*p*‐value 7.6 × 10^−100^), and GATA factors (*p*‐value 4.0 × 10^−67^), as well as DR‐1 element bound by PPAR nuclear receptors (*p*‐value 1.6 × 10^−10^, Figure 3e). The motifs for pioneer factors found in regions occupied by Foxa2 in *Zmpste24* mutants correspond to those detected in sites bound by lamin B1 and tethered to the nuclear lamina in young livers. We performed functional analysis of genes associated with Foxa2 binding sites in *Zmpste24*‐deficient mice using IPA. Analogous pathways are affected by Foxa2 binding in Zmpste24‐deficient livers as in older hepatocytes (Bochkis et al., 2014) (Figure 3f), including activation of genes regulated by PPARα and LXR, factors that contribute to steatosis in older livers. Functional categories are associated with activation of additional nuclear receptors (FXR and PXR, Figure 3f), which is consistent with our previous findings that ligand‐dependent activation of nuclear receptors requires Foxa2 (Bochkis et al., 2009).

### 2.3 Direct Foxa2 targets are upregulated in Zmpste24 mutants and contribute to fatty liver phenotype

**Figure 4:**
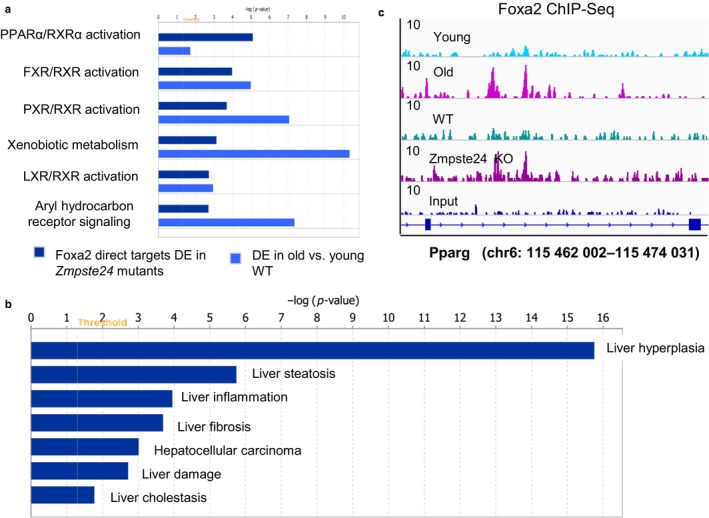
Direct Foxa2 targets are upregulated in *Zmpste24* mutants and contribute to fatty liver phenotype. (a) Comparison of overrepresented pathways identified by Ingenuity Pathway Analysis (IPA) between direct targets of Foxa2 in *Zmpste24*‐deficient mice and differentially expressed genes in aged WT livers (RNA‐Seq data from our previous study (Bochkis et al., 2014)). (b) Hepatotoxicity functions in Foxa2 direct targets identified by IPA. “liver hyperplasia” (*p*‐value 1.75 × 10^−16^) and “liver steatosis” (*p*‐value 1.80v10^−6^) are the most significantly enriched. (c) ChIP‐Seq track view of two novel Foxa2 sites in old hepatocytes and *Zmpste24* mutants in the intron of *Pparg*. Reads were merged from three replicates for binding in *Zmpste24* mutants and their control littermates. Data for young and old livers (merged from two replicates in each condition) are from our previous study (Bochkis et al., 2014). Size of the region is shown on *x*‐axis and magnitude of ChIP‐Seq signal is shown on *y*‐axis

To investigate whether Foxa2 binding is functional and influences gene expression, we focused on direct targets of Foxa2 in *Zmpste24*‐deficient mice, defined as bound and upregulated as Foxa2 is a transcriptional activator (Lai et al., 1990). Binding sites were mapped to 2,293 nearby genes using GREAT (McLean et al., 2010) and compared to gene expression profile of *Zmpste24* mutants (Osorio et al., 2010). We identified 814 direct targets (fold change >2), while expression of 118 genes near a Foxa2 site decreased (fold change <−2). Just like in comparison with all Foxa2‐binding sites (Figure 3f) in *Zmpste24*‐deficient livers, similar pathways were overrepresented in Foxa2 direct targets and differentially expressed genes in older hepatocytes (Bochkis et al., 2014) (Figure 4a), including activation of PPARα and LXR. Hepatotoxicity functions in Foxa2 direct targets identified by IPA included highly enriched “liver hyperplasia” (*p*‐value 1.75 × 10^−16^) and “liver steatosis” (*p*‐value 1.80 × 10^−6^) (Figure 4b). The full set of pathways for Foxa2‐bound regions and direct targets of Foxa2 is provided in Table S2. The direct targets included PPARγ, a nuclear receptor involved in development of fatty liver (Figure 4c) (Gavrilova et al., 2003). Hence, additional Foxa2 binding in *Zmpste24* mutants, like in old liver, leads to activation of gene expression that contributes to hepatic steatosis.

### 2.4 Foxa2 binding is correlated to loss of lamin B1 and H3K9me3 occupancy in old livers

**Figure 5:**
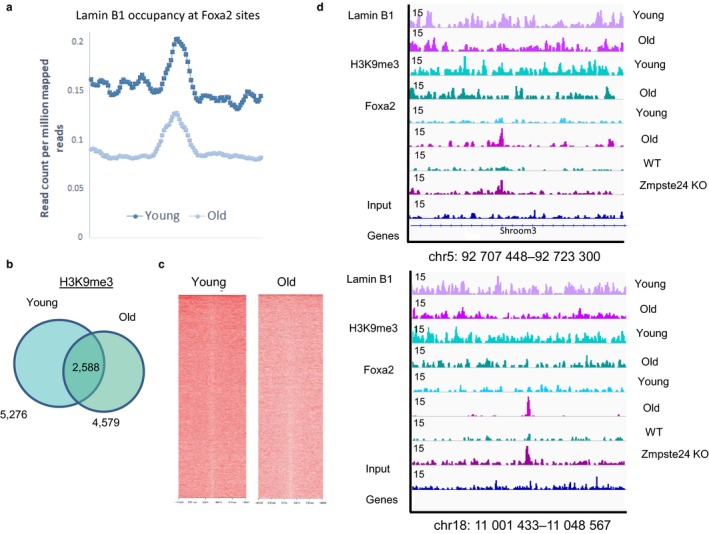
Regions where Foxa2 binding corresponds to decrease in lamin B1 occupancy. (a) Average profile of lamin B1 coverage (reads are normalized per million of mapped reads) in young and old livers at 4,434 sites bound by Foxa2 in old liver. Lamin B1 occupancy decreases in old livers. (b) Venn diagram showing the results of genomewide location analysis for H3K9 trimethylation in young and old liver, identifying 5,276 regions in young and 4,579 in old, of which 2,588 were called bound in at both ages. (c) Coverage plot of H3K9me3 signal in young and old livers for sites bound by Foxa2 in old livers in 5 kb regions surrounding the site. (d) Examples of regions where Foxa2 binding (in old livers and *Zmpste24* mutants) corresponds to a decrease in lamin B1 binding and levels of H3K9me3 in old livers (top: chr5:92,707,448‐92,723,300, bottom: chr18:11,001,433‐11,048,567). Data for young and old livers (merged from two replicates in each condition) are from our previous study (Bochkis et al., 2014) with input track as a comparison. Size of the region is shown on *x*‐axis and magnitude of ChIP‐Seq signal is shown on *y*‐axis

To investigate whether changes in occupancy by pioneer factor Foxa2 could have functional changes in old livers, we compared increased Foxa2 binding to changes in lamin B1 occupancy in old livers. We computed the overlap of all lamin B1 domains in young livers with Foxa2‐binding sites in old liver and found 4,434 such sites occupied by Foxa2. The correlation is significant (2,967 sites are found in a background set of random genomic regions of same size, Fisher's exact test *p*‐value <.0001). An average profile of lamin B1 occupancy in young and old livers for these 4,434 sites is shown in Figure 5a. Lamin B1 is substantially reduced at these sites. In addition, sum of lamin B1 coverage in 10 kb region surrounding the Foxa2 site is higher in young livers for 95% of the sites. These results suggest that even in large domains that are called bound by lamin B1 in both young and old livers, additional Foxa2 binding in old mice is accompanied by loss of lamin B1 occupancy locally.

Next, we mapped 4,434 Foxa2‐binding sites to closest genes using GREAT Chip‐Seq analysis tool (McLean et al., 2010) and computed an overlap with RNA‐Seq data set of differentially regulated genes in old livers from our previous study (Bochkis et al., 2014). Three hundred seventy‐five such genes were analyzed for enrichment using EnrichR, a collection of distinct gene set libraries (Kuleshov et al., 2016) (Figure S2). CheA library compared existing ChIP‐Seq data sets with our list of genes and identified binding sites for PPAR, LXR, and their heterodimer partner RXR significantly enriched in our gene set. Positional weight matrix analysis found the *forkhead* consensus, as well motifs for nuclear receptors. NCI‐Nature pathways implicated the Foxa2 transcription network. And WikiPathways database associated our gene list with adipogenesis targets. Collectively, enrichment analyses of Foxa2 targets bound in old livers in regions of lamin B1 loss corroborate our model. The overlap between these targets and genes differentially expressed in Zmpste24 mutants is provided in Table S3.

Furthermore, we related the changes in heterochromatin mark H3K9me3 (5,276 regions in young, 4,579 regions in old livers with overlap of 2,588, Figure 5b) to Foxa2 binding. For sites bound by Foxa2 in old livers, we plotted H3K9me3 coverage in young and old livers in 5 kb regions surrounding the site. We found a reduction in H3K9me3 levels, showing a correlation between reduced H3K9me3 signal and increased Foxa2 binding (Figure 5c). We show examples of genomic regions where Foxa2 binding corresponds to decrease in both lamin B1 binding and H3K9me3 occupancy in old hepatocytes, suggesting that these regions are no longer tethered to the lamina and closed and could be open to transcription (Figure 5d). In addition, Foxa2 binding in *Zmpste24* mutants resembles Foxa2 occupancy in old livers in these regions.

**Figure 6:**
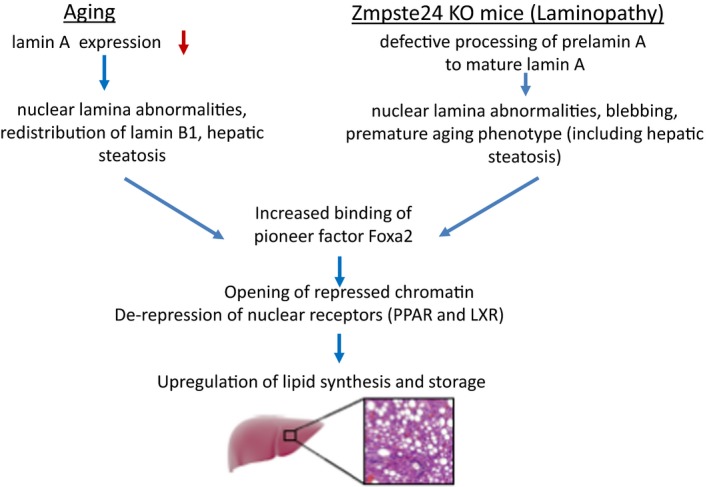
Changes at the nuclear lamina alter binding of pioneer factor Foxa2 leading to hepatic steatosis. Aged and *Zmpste24*‐deficient livers share several features, including nuclear lamina abnormalities, increased Foxa2 binding, de‐repression of PPAR‐ and LXR‐dependent gene expression, and fatty liver. In old livers, additional Foxa2 binding is correlated to loss of lamin B1 and heterochromatin (H3K9me3 occupancy) at these loci. Our observations suggest that changes at the nuclear lamina are linked to altered Foxa2 binding, enabling opening of chromatin and de‐repression of genes encoding lipid synthesis and storage targets that contribute to etiology of hepatic steatosis

In summary, we found that lamin B1 binds distinct domains in young and old livers. Sequences in sites bound by lamin B1 in young livers are enriched for the forkhead motif. We observed that protein levels of lamin A decrease during chronological aging in hepatocytes where binding of pioneer factor Foxa2 is increased. We found that Foxa2 occupancy is also increased in *Zmpste24* mutant mice, a laminopathy model, connecting our findings to Foxa2 binding in old livers. Aged and *Zmpste24*‐deficient livers share several features, including nuclear lamina abnormalities, increased Foxa2 binding, de‐repression of PPAR‐ and LXR‐dependent gene expression, and fatty liver. In old livers, additional Foxa2 binding is correlated to loss of lamin B1 and heterochromatin (H3K9me3 occupancy) at these loci. Our observations suggest that changes at the nuclear lamina are linked to altered Foxa2 binding, enabling opening of chromatin and de‐repression of genes encoding lipid synthesis and storage targets that contribute to etiology of hepatic steatosis (Figure 6).

## 3 DISCUSSION

Here, we used an unbiased approach to investigate changes in LADs in aging and are providing these data sets as a resource to the field. While this work is the first to connect regulation of chromatin at the nuclear lamina to binding of pioneer factors in mammalian aging and age‐dependent metabolic dysfunction, Mango and colleagues have reported that binding of pha‐4, an ortholog of Foxa proteins in *Caenorhabditis elegans,* is restricted by emerin, a nuclear lamina component, in the pharynx during foregut development. On this basis, they concluded that nuclear lamina interferes with binding of pha‐4, preventing global decompaction and reorganization of chromatin (Fakhouri, Stevenson, Chisholm & Mango, 2010). Collectively, these observations indicate that the relationship between lamina components and pioneer factors is conserved and warrants further investigation.

Both LAD sequences and *forkhead* motifs are A/T‐rich, suggesting a model where the nuclear lamina and pioneer factors could vie for genomic regions such as nucleosomes and transcription factors compete for DNA binding (Workman & Kingston, 1992). In this paradigm, pioneer factors would be prevented from binding when these loci are sequestered to the nuclear envelope but could access these sites once the chromatin is no longer tethered to the lamina.

While we find that A/T‐rich motifs, including the *forkhead* consensus, are significantly enriched in sites bound by lamin B1 exclusively in young livers and those shared between young and old livers, CG‐rich motifs are more prevalent in lamin B1 regions bound in old livers. Increased CG content is associated with DNA methylation. Analysis of DNA methylation in aged blood cells has demonstrated presence of age‐associated hypomethylated domains, which exhibit preferential hypomethylation in cancer independently of tissue of origin (Yuan et al., 2015). Another study has linked hypomethylated domains in cancer to LADs (Berman et al., 2012). Hence, the presence of CG‐rich motifs in LADs in old livers could indicate an age‐dependent dysregulation of DNA methylation in the liver.

We observe striking similarities between old and *Zmpste24* mutant livers, including nuclear abnormalities, changes in lamin A expression, and increased Foxa2 binding leading to de‐repression of PPAR‐ and LXR‐dependent gene expression that contributes to development of fatty liver. Hepatic steatosis is a common phenotype for aged mice (Jin et al., 2010; Ogrodnik et al., 2017), *Zmpste24* mutants (Marino et al., 2008; Pendas et al., 2002), and liver‐specific *Lmna* knockouts (Kwan et al., 2017). In addition, mutations in LMNA and ZMPSTE24 in patients lead to metabolic dysfunction, including type 2 diabetes and hepatic steatosis (Galant et al., 2016; Shackleton et al., 2000). Moreover, a recent report has implicated disorganization of heterochromatin at the lamina as a driver of human aging (Zhang et al., 2015). Numerous enzymes associated with H3K9me3, the mark associated with heterochromatin, including acetyltransferases (SRC‐1/*Ncoa1*), deacetylases (Hdac3), methyltransferases (Suv39 h1 and G9a/*Ehmt2*), and demethylases (Jhmdh2a/Kdm3a, Jmjd2c/Kdm4c), have been linked to fatty liver, diabetes, and obesity (Picard et al., 2002; Sun et al., 2012; Tateishi et al., 2009; Wang et al., 2013). In addition, levels of H3K9me3 increase during caloric restriction and decrease in *db/db* mice and other models of hyperglycemia (Vaquero & Reinberg, 2009; Villeneuve et al., 2008). Together, these studies underscore the importance of investigating how dysregulation of heterochromatin at the nuclear lamina leads to metabolic dysfunction.

## 4 EXPERIMENTAL PROCEDURES

### 4.1 Mice

Male mice (C57BL6) were purchased from the National Institute of Aging (NIA) aged rodent colony (Charles River Laboratories). Two biological replicates of young and old mice were used for chromatin immunoprecipitation and sequencing. The derivation of Zmpste24 null mice has been described previously (Varela et al., 2005). 12‐week‐old wild‐type control littermates and *Zmpste24* male mutants (three biological replicates each) were used for ChIP‐Seq experiments. All animal work was approved by MIT's Committee on Animal Care.

### 4.2 Immunofluorescence and immunohistochemistry

Indirect immunofluorescence and immunohistochemistry were performed as described previously (Zhang, Rubins, Ahima, Greenbaum & Kaestner, 2005). Slides subject to immunohistochemistry were counterstained with hematoxylin and eosin. Hoechst 33528 nucleic acid stain (ThermoFisher, 1:2,000) was used to detect nuclei.

### 4.3 Chromatin immunoprecipitation and ChIP‐Seq

Snap‐frozen mouse liver (100 mg) from wild‐type mice was used to prepare chromatin. ChIP and ChIP‐Seq were performed as reported previously (Bochkis et al., 2014). Briefly, liver tissue was minced in cold PBS and cross‐linked in 1% formaldehyde/PBS for 15 min with constant rotation in Labquake tube rotator. Cross‐linking was quenched by adding glycine to a final concentration of 0.125 m. Nuclear lysate was sonicated using Diagenode Bioruptor for 20 min (30 s on/30 s off). Libraries were made according to standard Illumina protocol (end repair of ChIP DNA, addition of A base to the 3′‐ends, adapter ligation, and amplification). We used multiplex adapters for sequencing and Kapa HiFi DNA polymerase (Kapa Biosystems) for PCR amplification (16 cycles). Library fragments were isolated using Pippin Prep agarose gel. The purified DNA was captured on an Illumina flow cell for cluster generation. Libraries were sequenced on Illumina HiSeq 2,000 and HiSeq 2,500 instruments following the manufacturer's protocols.

Foxa2‐specific rabbit antiserum (Seven Hills Bioreagents, WRAB‐1,200), rabbit antibody to Di‐Methyl‐Histone H3 Lys9 (Cell Signaling, D85B4), goat antibody to lamin B1 (Santa Cruz Biotechnology, sc‐6,216), and rabbit antihistone H3 (trimethyl K9) antibody (ab8898) were used for immunoprecipitation. Libraries were sequenced on an Illumina HiSeq 2,000 and HiSeq 2,500 instruments (40 bp and 60 bp single‐end reads, read summary in Table S4).

### 4.4 RNA and protein analysis

Liver RNA was isolated from young and old wild‐type mice, and quantitative reverse transcription–PCR was performed as described (Zhang et al., 2005). Protein extracts preparation and protein immunoblot analysis were performed as reported previously (Bochkis et al., 2008). The primary antibodies used were rabbit antibody to FOXA2 (Seven Hills Bioreagents, WRAB‐1,200, 1:5,000), rabbit antibody to histone H3 (Cell Signaling #4,499), goat antibody to LAMIN A/C (Santa Cruz, sc‐7,196, 1:100), goat antibody to LAMIN B (Santa Cruz, sc‐6,215, 1:100), rabbit antibody to LAMIN B (Abcam, ab16048), and rabbit antibody to TBP (Santa Cruz, sc‐273, 1:100). Protein expression was quantified by imagej software.

### 4.5 ChIP‐Seq analysis

Reads were aligned to the mouse genome (mm10; NCBI Build 38, Table S4) using BWA v0.7.12 (Li & Durbin, 2009). Duplicate reads were removed using Picard v 1.134 (http://picard.sourceforge.net). Reads (phred score ≥20) that aligned uniquely were used for subsequent analysis. Data from two biological replicates were merged for lamin B1 ChIP‐Seq comparison (two young and two old). Data from three biological replicates were merged for Foxa2 binding in Zmpste24 mutants (three WT and three KO). Epic peak caller (https://github.com/daler/epic), a more efficient implementation of SICER domain‐calling algorithm (Xu et al., 2014), was used to determine lamin B1‐associated domains (mm10 as species, window size of 10 kb, gap size of 3). PeakSeq (Rozowsky et al., 2009) was used to identify Foxa2 bound peaks against input controls (FDR 5%, q‐value = 0.12). Pairwise correlations among samples for regions at least 50 bp long were computed in R. We used input reads from the previous study (Bochkis et al., 2014), a mixture of input chromatin from young and old wild‐type livers (42.9 million total reads).

For Foxa2 ChIP‐Seq, as there were more reads in Zmpste24 mutants (21 million in WT, 31 million in KO, Table S4), KO reads were downsampled to 21 million. We used PeakSeq (21 million WT reads vs. input controls and 21 downsampled KO reads vs. input controls) to identify Foxa2 bound peaks. Epic/SICER performs normalization of data using a technique based on filtering with islands, as described in Zang et al. (2009), thus precluding the need for downsampling of lamin B1 ChIP‐Seq data (89 million reads in Young, 59 million reads in Old, Table S4).

### 4.6 Functional analysis

ChIP‐Seq peaks were associated with closest genes with the GREAT analysis (parameters: single closest gene, 10,000 kb) (McLean et al., 2010). Overlap between different categories of binding sites was computed using Galaxy genome analysis tools (Hillman‐Jackson, Clements, Blankenberg, Taylor & Nekrutenko, 2012). Sequencing reads were visualized with the Integrative Genome Viewer (IGV)(Robinson et al., 2011). Ingenuity Pathway Analysis (IPA) and PWM scan analysis were performed as reported previously (Bochkis et al., 2014). Reads were extended to the size of the library insert (150 bp for Foxa2) to obtain sequences for motif analysis. De novo motif analysis was completed using HOMER (findMotifsGenome.pl command, young vs. old (as background), old vs. young (as background)). For overlap analysis (Figure S1), for sites bound by Foxa2 in old livers, we computed coverage (using bedcov function in samtools) in alignment files for young (WT) and Zmpste24 mutant livers. We used a cutoff of 400 bases per region (or read base count/read length = read count, 400/75 = 5.3 reads per region). Heatmaps were constructed using ngs.plot software (Shen, Shao, Liu & Nestler, 2014).

### 4.7 Accession numbers

Genomic data from this study can be accessed at GEO under accession number GSE58006 (SubSeries GSE60393 for ChIP‐Seq) and GSE78177.

## CONFLICT OF INTEREST

None declared.

## AUTHOR CONTRIBUTIONS

H.W. performed experiments. L.N.S., M.A.P., and A.J.P. analyzed data. F.G.O. and C. L. O. contributed to studies with *Zmpste24* mutant mice. I.M.B. developed the project, performed experiments, analyzed data, and wrote the draft of the manuscript.
