# Supplementary material for: Changes at the nuclear lamina alter binding of pioneer factor Foxa2 in aged liver
Source: Aging Cell. 2018 Feb 27;17(3):e12742. doi: 10.1111/acel.12742 (PMC5946061; doi:10.1111/acel.12742)
Supplement: Supplementary file 1 [file ACEL-17-e12742-s001.pdf]

Figure S1

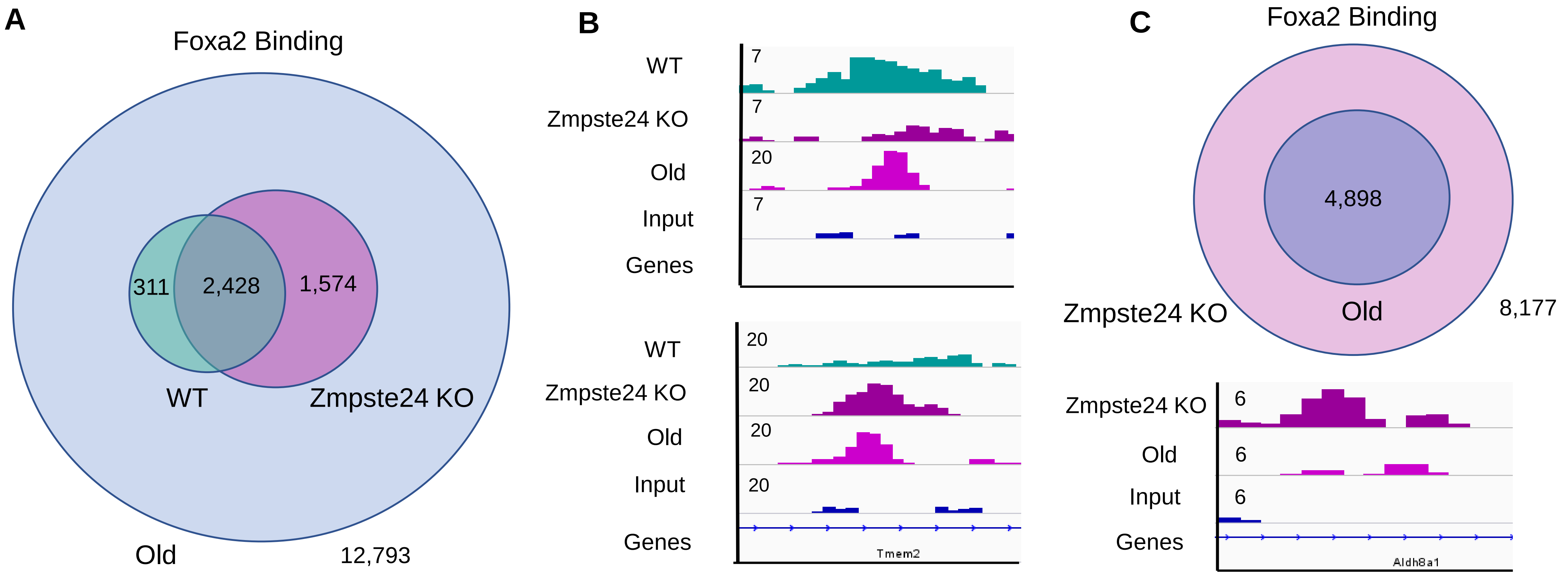
A
Foxa2 Binding
311
2,428
1,574
WT
Zmpste24 KO
Old
12,793
B
WT
Zmpste24 KO
Old
Input
Genes
7
7
20
7
WT
Zmpste24 KO
Old
Input
Genes
20
20
20
20
Tmem2
C
Foxa2 Binding
4,898
Zmpste24 KO
Old
8,177
Zmpste24 KO
Old
Input
Genes
6
6
6
Aldh8a1

Figure S2

ChEA 2016

Index	Name	P-value
1	RXR_22158963_ChIP-Seq_LIVER_Mouse	7.334e-19
2	LXR_22158963_ChIP-Seq_LIVER_Mouse	7.334e-19
3	CLOCK_20551151_ChIP-Seq_293T_Human	4.009e-9
4	PPARA_22158963_ChIP-Seq_LIVER_Mouse	4.785e-11
5	MYB_21317192_ChIP-Seq_ERMYB_Mouse	8.331e-7
6	ESR2_21235772_ChIP-Seq_MCF-7_Human	0.00005844
7	RELA_24523406_ChIP-Seq_FIBROSARCOMA_Human	0.00001207
8	SALL1_21062744_ChIP-ChIP_HESCs_Human	0.003695
9	ESR1_21235772_ChIP-Seq_MCF-7_Human	0.004061
10	KDM2B_26808549_Chip-Seq_SUP-B15_Human	0.00002989

WikiPathways 2016

Index	Name	P-value
1	Translation Factors_Homo sapiens_WP107	0.002643
2	mRNA processing_Mus musculus_WP310	0.003746
3	Adipogenesis genes_Mus musculus_WP447	0.002928
4	Fluoropyrimidine Activity_Homo sapiens_WP1601	0.003270
5	Adipogenesis_Homo sapiens_WP236	0.003221
6	NRF2 pathway_Homo sapiens_WP2884	0.006461
7	Spinal Cord Injury_Homo sapiens_WP2431	0.007287
8	mRNA Processing_Homo sapiens_WP411	0.01025
9	Spinal Cord Injury_Mus musculus_WP2432	0.009531
10	Neural Crest Differentiation_Homo sapiens_WP2064	0.01206

NCI-Nature 2016

Index	Name	P-value
1	FOXM1 transcription factor network_Homo sapiens_c51cda49-6192-11e5-8ac5-06603eb7f303	0.0008760
2	FOXA1 transcription factor network_Homo sapiens_aa3927b7-6192-11e5-8ac5-06603eb7f303	0.001360
3	FOXA2 and FOXA3 transcription factor networks_Homo sapiens_b6933be8-6192-11e5-8ac5-06603eb7f303	0.009984
4	Retinoic acid receptors-mediated signaling_Homo sapiens_5797691b-6195-11e5-8ac5-06603eb7f303	0.01525
5	ALK1 signaling events_Homo sapiens_fcc72679-6186-11e5-8ac5-06603eb7f303	0.01116
6	Validated targets of C-MYC transcriptional repression_Homo sapiens_6bbdafa6-6196-11e5-8ac5-06603eb7f303	0.03074
7	AP-1 transcription factor network_Homo sapiens_3ce2f9c5-6189-11e5-8ac5-06603eb7f303	0.04092

TRANSFAC and JASPAR PWMs

Index	Name		P-value
1	NFYB (human)		0.0002213
2	TCFAP2A (human)		0.002096
3	NKX3-1 (human)		0.01167
4	CRTC3 (human)		0.009013
5	MAPK14 (human)		0.01011
6	CREB1 (mouse)		0.01039
7	RXRA (human)	Nuclear Receptor	0.01144
8	XBP1 (human)		0.02090
9	RARA (human)	Nuclear Receptor	0.02881
10	FOXO3A (human)	Forkhead	0.03436
