# Supplementary material for: Changes at the nuclear lamina alter binding of pioneer factor Foxa2 in aged liver
Source: Aging Cell. 2018 Feb 27;17(3):e12742. doi: 10.1111/acel.12742 (PMC5946061; doi:10.1111/acel.12742)
Supplement: Supplementary file 5 [file ACEL-17-e12742-s005.docx]

**Table S4** Sequencing statistics of ChIP-Seq libraries

ChIP-Seq library statistics (40 bp & 60bp single-end) showing aligned reads for H3K9me2, H3K9me3, and lamin B1 binding in young and old livers (pooled for two replicates in each condition) and Foxa2 binding in wildtype and *Zmpste24* mutants (pooled for 3 replicates in each condition). Input reads are a mix of Input from young and old wildtype livers.

* For PeakSeq analysis of Foxa2 binding, reads from *Zmpste24* KO were downsampled to 20,937,091 for comparison with WT samples

| **ChIP** | **Young** | **Old** |
| --- | --- | --- |
| **H3K9me2** | 25,443,375 | 24,238,479 |
| **H3K9me3** | 81,831,873 | 143,802,628 |
| **Lamin B1** | 88,943,202 | 59,340,254 |

| **ChIP** | **WT** | ***Zmpste24* KO** |
| --- | --- | --- |
| **Foxa2** | 20,937,091 | 31,243,764* |

| **Input** | 42,920,043 |
| --- | --- |
